# Pharmacological Effects of Genistein on Cardiovascular Diseases

**DOI:** 10.1155/2023/8250219

**Published:** 2023-05-26

**Authors:** Shima Jafari, Melika Shoghi, Mohammad Reza Khazdair

**Affiliations:** ^1^Cardiovascular Diseases Research Center, Birjand University of Medical Sciences, Birjand, Iran; ^2^Department of Clinical Pharmacy, School of Pharmacy, Birjand University of Medical Sciences, Birjand, Iran; ^3^Student Research Committee, Birjand University of Medical Sciences, Birjand, Iran

## Abstract

Cardiovascular diseases (CVDs) are a group of disorders that involve the heart or blood vessels and are the leading cause of mortality worldwide. Natural products have several pharmacological activities, such as anti-inflammatory, antioxidant, and immunoregulatory properties. This review summarizes the possible therapeutic effects of Genistein on CVD. The information from the current review study was obtained by searching for the keywords such as “Genistein”, “Cardiac dysfunction”, “hypertrophy”, and “Ischemia” “lipid profile” in different online database such as PubMed, Scopus, and Google Scholar, until February 2022. The results of the studies showed that genistein intake has a promising effect on improving cardiac dysfunction, ischemia, and reperfusion of the heart, decreasing cardiac toxicity, modulating lipid profile, and lowering blood pressure. The preventive effects of genistein on experimental models of studies were shown through mechanisms such as anti-inflammatory, antioxidant, and immunomodulatory effects. Pharmacological effects of genistein on cardiac dysfunction, cardiac toxicity, lipid profile, and hypertension indicate the possible remedy effect of this agent in the treatment of CVD.

## 1. Introduction

Cardiovascular diseases (CVDs) are a group of disorders of the heart and blood vessels that lead to the cause of mortality worldwide, approximately 31% of all global deaths [1]. CVDs consist of many types of diseases such as coronary heart disease (CHD), acute coronary syndrome (ACS), heart failure, stroke, cardiomyopathies, peripheral vascular diseases, hypertension, and dyslipidemias [2].

Diabetes, dyslipidemias, hypertension, smoking, lack of physical activity, and obesity are modifiable cardiovascular-related risk factors [3]. Prevalence of CVDs can be decreased by recognition and prevention of these risk factors. Besides lifestyle modifications, antihypertensive drugs, lipid lowering, and antithrombotic therapies are the main regimes for the prevention and treatment of CVDs [4].

For thousands of years, plants have been used for medicinal purposes and as an origin of the new drug discoveries which help the management of disorders. For example, aspirin, digoxin, and lovastatin are extracted from *salix alba* L. tree, *digitalis purpurea*, and *Monascus purpureus*, respectively [5, 6].

Phytoestrogens are a class of plant which has similar structures to estrogen, which allows them binding to estrogen receptor. Coumestans, isoflavones, and lignans are the main classes of phytoestrogens [7, 8].

Genistein is a phytoestrogen that belongs to the isoflavones family of Leguminosae plants. Genistein is a good choice for the management of some diseases because of its pharmacological and physiological properties [9]. Also, due to the pharmacological and biological properties such as antioxidant, anti-inflammatory, and anti-infective effects, isoflavones are considered for therapy/prevention purposes in medicine [10–12].

Genistein selectivity to estrogen receptor *β* is 20 times more than *α*, which is an acceptable substitute for estrogen replacement in postmenopausal women [13]. Genistein has both estrogenic and antiestrogenic activities by binding to estrogen receptor and sex hormone-binding proteins [14]. Genistein consumption reduces the incidence of prostate and breast malignancy by decreasing the testosterone level and competition with natural estrogen and, finally, inhibition of cancer cell growth and tumor development [15, 16].

The notable effect of genistein on bone density and bone turnover makes it as a principal option for the prevention of osteoporosis [17]. Genistein has safety and tolerability and has no significant change on liver function and hematology test result, and it could be a good reason to use genistein as medicine [8].

Previous studies revealed that dietary intake of genistein was related to lesser risk of cardiovascular diseases; therefore, it is time to assess the evidence of various effects of genistein on cardiovascular diseases. So, evaluating the effects of genistein on cardiovascular disease is the purpose of this systematic review.

## 2. Method

**Figure 1:**
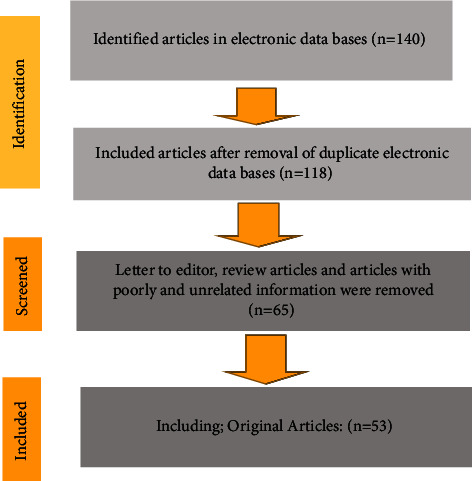
Flowchart of the process for selecting studies for the review.

The information of this study was obtained by searching the keywords such as “Genistein”, “Cardiac dysfunction”, “hypertrophy”, and “Ischemia” “lipid profile” in different online database such as PubMed, Scopus, and Google Scholar until February 2022. Articles published in languages other than English were excluded. A total of 140 articles were selected from the above databases. 22 articles were duplicates, 65 including; reviews, letter to editor, and some articles with poorly and unsuitable information were removed, and at least 53 original articles related to the topic of this review were included in the current study as shown in Figure 1.

## 3. Results

### 3.1. Genistein Effects on Cardiac Dysfunction and Ischemia/Reperfusion

#### 3.1.1. In Vitro Studies

The cardioprotective effect of genistein against cobalt chloride (CoCl2) induced hypoxia in H9c2, up-regulated the expression of Notch-1, reduced cell death, and suppressed HIF-1*α* expression. Furthermore, genistein could antagonize CoCl2-induced apoptosis through inhibiting caspase-3 expression and up-regulating Bcl-2/Bax ratio [18].

Genistein treatment (10^−5^ to 10^−10^ *μ*mol) in an acute myocardial ischemia model, increased the endothelial colony-forming cell (ECFC) migration and proliferation, and parallel increased the expression of ILK, *α*-parvin, F-actin, and phosphorylation of ERK 1/2 signaling. ECFCs induction into myocardial ischemic sites, cellular expansion stimulation, and angiogenic modulators secretion at the damaged tissues are the result of genistein treatment. Genistein also improved cardiac function, neovascularization, and decreased myocardial fibrosis. The genistein administration to ECFCs before transplantation improved the regenerative potential in ischemic tissues; therefore, this is a new approach for stem cell therapy in ischemic diseases [19].

Treatment of isoproterenol (ISO)-induced H9c2 cardiomyoblast cells with genistein suppressed the mitochondrial pro-apoptotic proteins expression (Bad, caspase-3, caspase-8, and caspase-9). Also in treated cells, genistein expressed many survival proteins such as phosphor p-Bad, (p)-Akt, and p-Erk1/2. Furthermore, through the expression of NF*κ*B, Akt, and Erk1/2 proteins, genistein has a protective effect [20]. These studies showed that genistein is a natural and safe selective estrogen receptor modulator (SERM) substitute to hormone replacement in cardioprotection.

Genistein outcome on lipopolysaccharide (LPS)-induced myocarditis in H9c2 cells enhanced the potential of cells growth and prevented cells apoptosis and inflammatory reaction. The results also showed that MAPK as the signaling pathway and Myc (a well-target gene of genistein) as the potential target of genistein in myocarditis [21].

Genistein protective effects against oxidative stress on cardiomyoblasts (H9C2) were studied. Treatment of cells with genistein (10 pM and 100 nM; for 24 h) modulated NO release, induced endothelial NOS-dependent NO production through modulation of intracellular signaling related to Akt, p38MAPK, and ERK1/2, increased the content of glutathione, and decreased reactive oxygen species production [22].

#### 3.1.2. In Vivo Studies

Intraperitoneal administration of genistein (5 mg/kg) showed cardioprotective effects in both sexes of rats. It decreased postischemic contractile recovery (PICR) and pressure/total heat rate (P/Ht) during low-flow ischemia/reperfusion (LFI)/R. Genistein also improved the heart dysfunction by Na^+^/Ca^2+^-exchanger (NCX) blockade with mitochondrial permeability transition pore (mPTP) opening [23].

Genistein administration (250 mg/kg) in ovariectomized (OVX) rats increased contractility (259 mm Hg/sec) and cardiac output (7 mL/min) above baseline. Genistein-treated hearts (*n* = 5) demonstrated ischemic tolerance, greater cardiac output, and markedly improvements of contractility after reperfusion. Also, genistein decreased mean glucose transporter protein 4 content [24].

Once daily injection of genistein (0.1 and 0.2 mg·kg) in male, Wistar rats showed that genistein prevents increase in heart weight to body weight ratio induced by isoproterenol, fibrosis, left ventricular mass, myocardial oxidative stress, myocyte size, and myocardial 1-OH proline. Inducible nitric oxide synthase (iNOS) inhibition and improvement of endothelial nitric oxide synthase (Enos) activities are the important effects of genistein on isoproterenol-induced cardiac hypertrophy [25].

Intraperitoneal administration of genistein improved pressure and left ventricular pressure/total heat rate in aged female and young male rats but not in young female rats in stunning induced by ischemia/reperfusion (I/R) in isolated hearts. In young male rats, genistein and estradiol have synergistic cardioprotective effect. After ischemia, SR Ca leak causing diastolic contracture was increased due to genistein. Results suggested that genistein was more cardioprotective and synergistic with estradiol, which depends on the mKATP and protein-kinase C channel pathway activation [26].

Administration of genistein (10, 50, and 100 mg/kg/day) on pressure overload-induced cardiac to mice markedly attenuated fibrosis hypertrophy and fibrosis 8 weeks later [27].

TGF*β*1-induced proliferation, myofibroblast transformation, and production of collagen on cardiac fibroblasts (CFs) were inhibited by genistein (20, 50, and 100 *μ*M) administration. Genistein also decreased the expression of TGF*β*-activated kinase 1 (TAK1) and induced antifibrotic effects. It also increased estrogen-dependent expression of metastasis-associated gene 3 (MTA3), which was a negative modulator of TAK1 [27].

The effects of genistein (as tyrosine kinase inhibitor) on hypertrophy induced by phenylephrine in ventricular myocytes cultured from neonatal rats prevented activation of three promoters, such as atrial natriuretic factor (ANF), Fos, and the myosin light chain 2 (MLC-2) in induced cells. Inhibition of the GTP loading of the Ras protein and activation of the mitogen-activated protein (MAP) kinases Erk1 and Erk2 in phenylephrine-induced cells were another effect of genistein [28].

The results demonstrated that for the activation of the Ras-MAP kinase pathway, which is a critical pathway in hypertrophic response regulation, genistein is important.

Genistein effects on pathological cardiac hypertrophy in mice model showed 7 weeks administration of genistein (40 mg/kg/day) significantly increased collagen volume fraction, cross-sectional area of cardiomyocytes, up-regulated expression of beta-myosin heavy chain (*β*-MHC) promoter, atrial natriuretic peptide (ANP), collagen I*α*, fibronectin, collagen III, TGF-*β*1, and connective tissue growth factor (CTGF) [29].

In pulmonary hypertension (PH) rats, genistein (1 mg/kg/day) administration showed decreased right ventricular ejection fraction to 41.99 ± 1.27% and increased peak systolic right ventricular pressure to 66.35 ± 1.03 mm Hg. Similar to the control group, right ventricular pressure was reduced, and right ventricular ejection fraction was completely restored to 65.67 ± 1.08%. Proliferation of the smooth muscle cell of the human pulmonary artery through estrogen receptor-*β* was inhibited by genistein [30].

Genistein (0.075 mg/min) infusion in pigs increased coronary blood flow by about 16.3%. Also, genistein induced NO production through p38 mitogen-activated protein kinase (MAPK), ERK 1/2, and Akt pathways. In addition, genistein caused coronary vasodilation via release of NO by vasodilatory beta (2)-adrenoreceptor [31].

In hypersensitive necrotic area, genistein IV administration attenuated the necrosis of myocardia and myocardial myeloperoxidase activity (MPO). It also decreased serum levels of TNF-*α*, creatinine phosphokinase activity (CPK), and ventricular arrhythmias and slowed intercellular adhesion molecule-1 (ICAM-1) expression in the injured myocardium [32]. These data suggested that myocardial ischemia-reperfusion injury protection and inflammatory response inhibition are some effects of genistein.

Genistein administration as dietary supplement (54 mg/day) in postmenopausal women for one year showed a significant development of left ventricular ejection fraction (LVEF) and left atrial (LA) area fractional change that followed 1 year of treatment. LA longitudinal strain peak and body surface area indexed LA systolic volume were changed by genistein. In women with metabolic syndrome (MetS), genistein intake (54 mg/day/year) improved both LA remodeling and function and LV ejection fraction [33]. Administration of dietary intake of the genistein in humans showed that genistein mitigates ischemic stroke-induced damage by alterations in molecular pathways. Genistein treatment blocked targeting estrogen, nuclear factor (NF)-kappa B, direct antioxidant action, androgen-mediated molecular, and Akt signaling pathways which are important for stroke damages decreasing and cell survival increasing [34]. Subcutaneous genistein administration of 0.2 mg/kg/day in ovariectomized New Zealand white female rabbits did not have a protection effect on ischemic myocardium in both nonovariectomized or ovariectomized animals for 4 weeks [35]. Compared to control groups, genistein treatment (600 mg/kg, diet) for 4 weeks in both sexes of C57BL/6J mice made echocardiographic changes in function, increased left ventricle internal dimension (LVIDs), cardiac GLUT4 protein expression, and significantly decreased fractional shortening and whole heart surface area [36].

Genistein effects on cardiac dysfunction and ischemia/reperfusion are shown in Table 1.

### 3.2. Genistein Effects on Induced Cardiac Toxicity

#### 3.2.1. In Vitro Studies

Dietary intake of genistein effect (20 microM) on ventricular myocytes in high glucose medium induced diabetic cardiomyopathy in adult rats reduced cardiac mechanical dysfunction, so it concluded that genistein had a significant effect against cardiac dysfunction induced by diabetes [38].

#### 3.2.2. In Vivo Studies

Administration of genistein in endotoxic shock guinea pig model in the chronic and acute treatment part (s.i And i.p, respectively) showed that genistein elevated the myocardial guanosine 3′,5′-cyclic monophosphate (cGMP) and concentration of plasma nitrate after 6-hour endotoxic shock. Genistein significantly attenuated the cardiac action potential duration (APD) shortening in endotoxic shock by lowering the plasma nitrate and the cardiac cGMP production in both kinds of administration [39].

The protective effects of genistein (5 mg/kg) against cardio toxicity induced by doxorubicin (DOX) in the mice model significantly reduced cardiac troponin and redox markers such as lipid peroxidation (LPO) and reactive oxygen species (ROS) in serum that increased by DOX. In addition, genistein reduced the expressions of interleukin (IL)-6, IL-8, and tumor necrosis factor (TNF)-*α* which increased during DOX-induced inflammatory and regulated antioxidant response by increased protein expressions of Nrf-2, HO-1, nitroquinoline oxide (NQO1) [40].

Oral administration of genistein (300 mg/kg/day) in streptozotocin (STZ)-induced diabetic rats showed decreasing in C-reactive protein, HbA1c, blood glucose, and expression of TGF-*β*1 and NF-*α* proteins that were increased in STZ-induced diabetes. In addition, compared to control groups, genistein treatment increased total antioxidant reserves of the hearts and reduced the ultrastructural degenerative changes in the cardiac tissues [41]. These results indicated that genistein had an anti-inflammatory and antioxidant effect.

Intraperitoneal injection of genistein (0.25, 0.5, or 1 mg/kg) in mice revealed cardioprotective and antinitrative/oxidative effects after burning injury in mice. Genistein reduced myocardial injury such as improved left ventricle ejection fraction, reduced creatine kinase levels, and lactate dehydrogenase in the serum. It also reduced expressions of inducible nitric oxide (NO) synthase, NO and superoxide anions production, and reduced peroxynitrite. Treatment with genistein significantly up-regulated the expression of the enhancer of split (Hes-1) and Notch-1 intracellular domain (NICD1) after burning injury [42]. Genistein effects on cardio toxicity are summarized in Table 2.

### 3.3. Effects of Genistein on Lipids Profile

#### 3.3.1. In Vitro Studies

Treatment of HepG2 cells cultured with genistein (0, 0.01, 1.00, 10.00, and 50.00 *µ*M) for 24 hr, decreased the secretion of apolipoprotein A1 (Apo-A1) and high-density lipoprotein-cholesterol (HDL-C), and, meanwhile at dose 1.00 *µ*M, increased total cholesterol (TC). Also, genistein upregulated 3-hydroxy-3-methyl glutaryl coenzyme A reductase (HMGCR), mRNA levels of low-density lipoprotein receptor (LDLR), and the protein and mRNA levels of sterol regulatory element binding proteins-2 (SREBP-2) at dose dependently and decreased peroxisome proliferator-activated receptor-*γ* (PPAR*γ*) mRNA level and liver *X* receptor (LXR*α*) [43]. These results indicated that genistein could inhibit cellular cholesterol efflux.

Genistein (1000 nM) administration in human umbilical vein endothelial cells (HUVECs) for 30 min showed inhibition of ox-LDL-induced lowering of the activity of SA-*β*-gal levels and P16 and P21 proteins. The genistein effect was bound up with elevating autophagic flux [44].

#### 3.3.2. In Vivo Studies

L-carnitine and genistein (50 mg/kg) administration on serum lipid and cytokine profiles in induced nephrotic syndrome demonstrated genistein effect on cardiovascular diseases by inducing alterations in metabolism of lipid and production of cytokine. In addition, after treatment with genistein, low-density lipoproteins (LDL) and interleukin-6 markedly decreased. The effect of genistein on triglyceride and high-density lipoproteins (HDL) levels was less than L-carnitine [45].

Genistein (20 mg/kg) administration for 42 days on polycystic ovary syndrome (PCOS) rats significantly increased insulin level. Genistein also significantly lower the levels of MDA TNF-*α* and higher SOD activity in the serum. Furthermore, in the histopathological analysis, genistein led to fewer cysts development and increase in luteinization [46]. Compare to control groups, estradiol (E2) and genistein (Gen) administration to ovariectomized (OVX) rats showed that treatment with E2 had significantly increased LDL chol, total cholesterol (Total-C), total oxidant status, and oxidative stress index. Also, in the genistein group, LDL chol and total-C decreased more than in the E2 treatment group. So we could conclude that Gen treatment might be preferred to E2 treatment for alleviating the menopausal symptoms and those who are at risk for cardiovascular diseases [47].

Genistein (600 mg/kg diet) supplement and soy protein (200 g/kg diet) in STZ-induced diabetic rat increased the glucokinase level and plasma insulin level but decreased glucose-6-phosphatase, thiobarbituric acid reactive substances, and HbA(IC) level of the STZ-induced diabetic rats. This combination administration significantly increased hepatic SOD, glutathione peroxidase, and catalase activities compared to the control groups [48].

Administration of 200 ppm genistein in rats by a 4-week feeding decreased cholesterol levels and improved calcium absorption efficiency. In addition, in OVX rats receiving an AIN-93M diet supplement with 200 ppm genistein, restored total and trabecular bone mineral density at the distal femur similar to the levels of sham rats [49].

Subcutaneous administration of Gen (10 mg/kg) in orchidectomized (Orx) and intact (IA) middle-aged male rats significantly reduced lipoprotein cholesterol and TC levels. Gen showed significant cholesterol-lowering effect but increased total triglycerides only in the Orx group [50]. Dietary of genistein on induced hyperlipidemia in male hamsters showed genistein (2 g/kg) significantly lowered plasma cholesterol, triglyceride, LDL cholesterol, MDA, and liver cholesterol than those in the high-fat diet (HFD) group. In addition, genistein significantly up-regulated the expression of *α* and *β* mRNA estrogen and hepatic LDL receptors compared to the HFD group [51].

Administration of 250 *μ*mol/L genistein that dissolved in dimethyl sulfoxide in humans showed that by decreasing lipid elements, genistein has beneficial effects on atherosclerotic risk and lowering platelet aggregation. Genistein decreased collagen- and ADP-dependent platelet activation and inhibited agonist-induced platelet aggregation by dose-dependent manner [52]. In a human study, administration of Gen (0.5–2.5 microM) inhibited Cu (^++^)-induced lipid peroxidation (LP) of HDL and increased the levels of conjugated dienes in lipoproteins oxidized. In addition, genistein blocked the alterations of the structure and physico-chemical properties of apoprotein, related to Cu (^++^) triggered LP of lipoproteins [53].

Supplementation with genistein (90 mg/day), vitamin D, and calcium in healthy postmenopausal women in clinical double blind study indicated variant allele rs9340799, rs928554, and rs4986938, and estrogen receptors (ERs) polymorphisms were associated with a greater reduction in LDL cholesterol, total cholesterol, and triglyceride levels. These results indicated the relationship between ER genes polymorphisms and improvement in the serum lipid profile after treatment with genistein in postmenopausal women [54].

The results of a placebo-controlled trial on postmenopausal women (*n* = 389) with low bone mass showed treatment with of genistein aglycone (54 mg) for 24 months significantly declined fasting glucose and insulin, homocysteine, and fibrinogen. Genistein did not affect HDL cholesterol and triglycerides through serum osteoprotegerin. These results showed that a healthy diet and treatment with genistein aglycone plus calcium showed beneficial effects on some CVD risk factors and homocysteine levels in low bone mass postmenopausal women [55].

Treatment of hyperlipidemic postmenopausal women in China with genistein (60 mg/day) for six months significantly lowered TC, TG, and LDL cholesterol levels, while HDL cholesterol levels as well as mRNA expression and protein levels of LDLR, and liver *X* receptor *α* (LXR*α*) were markedly increased [56]. The effects of genistein on lipids profile are shown in Table 3.

### 3.4. The Effects of Genistein on Hypertension

#### 3.4.1. In Vitro Studies

The effects of genistein on the vascular calcification in rat monocytes, osteoblasts cultures, and aortic vascular cell (*in vitro*) were evaluated. The mRNA expression of intercellular adhesion molecule-1 was reduced in the cells exposed to genistein (10 nM or 1 *μ*M). Also, the cell proliferation and migration on vascular muscle cells were also markedly reduced.

Both osteoblastic markers such as deposition of calcium nodules and alkaline phosphatase activity were markedly reduced after genistein treatment [57].

The acute treatment of thoracic aortae was isolated from adult male SHR rats with genistein (10 *μ*M) restores the impaired endothelial functions in the aorta.

Genistein transactivates epidermal growth factor receptor (EGFR) through membrane ER*α* via G protein-coupled pathways [58]. This is enhanced eNOS phosphorylation and hence endothelial function in the aorta. Genistein treatment (50 micromol/l) inhibited the expressions of p22phox NADPH oxidase subunit and angiotensin II type 1 receptor (AT1) in aortic endothelial cells from stroke-prone SHR. Genistein also significantly reduced the angiotensin II induced superoxide by reduction of nitroblue tetrazolium, endothelin-1 production, and inhibition of nitrotyrosine formation [59].

#### 3.4.2. In Vivo Studies

The effects of genistein (0.1–30 micromol/L) on aortic smooth muscle cells (SMC) from hypertensive rats showed inhibitory effects on proliferation and DNA synthesis of SMC in a dose-dependent manner. It also showed inhibitory effects on platelet-derived growth factor (PDGF) induced aortic SMC proliferation [60]. These results indicated that genistein treatment may be useful for reducing cell proliferation that involved in atherosclerotic vascular changes. Administration of genistein for 10 days (1 mg/kg) significantly improved lung and heart function by reduction in right ventricular pressure and restored right ventricular ejection fraction similar to the control group. Genistein also reversed pulmonary hypertension (PH)-induced pulmonary vascular remodeling (*in vivo*) and inhibited human pulmonary artery SMC proliferation (*in vitro*) likely through treatment with estrogen receptor-*β*. It also reversed right ventricular (RV) hypertrophy, inhibited neonatal rat ventricular myocyte hypertrophy, and restored PH-induced loss of capillaries in the RV [30]. The results indicated that genistein prevents the progression of severe PH to right heart failure.

Treatment of fructose-fed hypertensive rats with genistein (1 mg/kg) lowered blood pressure (BP), restored angiotensin-converting enzyme (ACE), protein kinase C-*β*II (PKC-*β*II), and expression of endothelial NO synthase (eNOS), and preserved renal ultrastructural integrity [61].

Effects of genistein on pulmonary arterial hypertension (PAH) in monocrotaline-induced rat model were also studied. Genistein (20, 80, and 200 *μ*g/kg) significantly improved diameter of pulmonary artery, speed of tricuspid regurgitation, mean pulmonary artery pressure, and RV hypertrophy index. Treatment with genistein also improved proliferation of smooth muscle, stenosis of pulmonary artery, RV hypertrophy, and myocardial hypertrophy. In rat lung tissue, phosphorylated protein kinase B (P-Akt), phosphorylated eNOS, and expressions of nitric oxide (NO) were remarkably increased in the genistein treatment group compared to the PAH group [62].

The genistein fed diet (600 mg/kg food) remarkably less weight than male's mice fed the genistein-free diet (0G). The Genistein fed diet also exhibited remarkably elevated serum insulin and decreased serum glucose levels. Basal vascular reactivity of isolated aortic rings (IAR) was increased remarkably by the genistein diet in both males and females mice [63].

Administration of genistein (1 mg/kg) on lipopolysaccharide (LPS)-induced rat cardiovascular abnormalities causes to reduction in contractile response to noradrenaline (NA) in vascular tissue, and inhibition in hypo-responsiveness to NA, increasing in nitrite plasma levels, and iNOS expression production by endotoxin. In addition, genistein restored lipid peroxidation, impaired aortic relaxation to acetylcholine, and suppressed long-term hypotension [64]. This result indicated that genistein prevented vascular alterations and hypotension induced by LPS that was mediated by its anti-inflammatory and antioxidant effects as well as inhibitory effect on NO overproduction. The effects of 10 nM genistein in rat aortic strips significantly increased nitric oxide synthesis. It also exhibited an antiaggregatory action, dependent on the NO release from vascular tissue, and suppressed the inhibition of platelet aggregation. Moreover, genistein inhibited platelet aggregation in aortic strips from ovariectomized rats [65].

The vascular effects of genistein in ovariectomized spontaneously hypertensive rats (SHR) were evaluated. Animals treated with genistein (2.5 and 25 mg/kg), for 2 days or 2 weeks, and contractility of the renal arterial rings were studied. The tissue contractions and tyrosine phosphorylation were reduced by the genistein (2.5 mg/kg) for 2 days [66].

Genistein treatment (20 and 80 *µ*g/kg) on heme oxygenase-1 (HO-1) expression in PAH induced by monocrotaline (MCT) in rats as dose dependently improved the RV hypertrophy index, increased the expression of HO-1, and decreased the elevated mean PA pressure [67].

Dietary genistein (0.06%, wt/wt) decreased NaCl-sensitive hypertension in young, male stroke-prone spontaneously hypertensive rats (SHRs). Genistein also declined plasma insulin and insulin resistance in SHR on a high NaCl diet and reduced plasma triglycerides and cholesterol in SHR. The antihypertensive effect of genistein was influenced by the autonomic nervous system [68].

**Figure 2:**
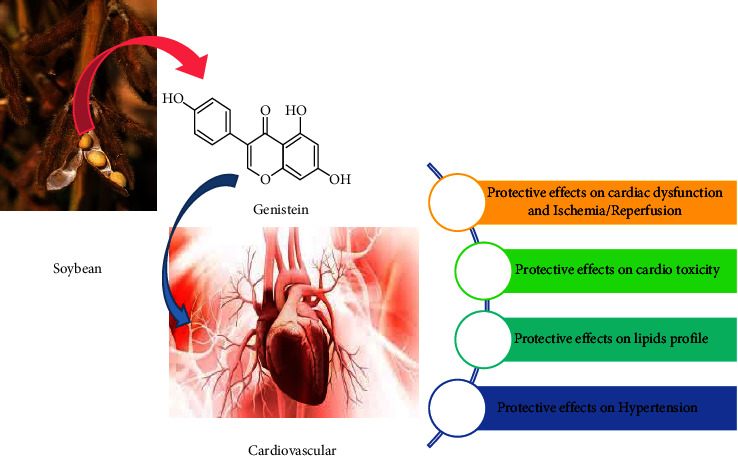
Possible therapeutic effects of genistein on cardiovascular diseases.

Treatment of 23-week-old rats with genistein (10 mg kg) reduced NADPH-induced O_2_ (^−^) production and systolic blood pressure (SBP) and enhanced endothelium-dependent aortic relaxation to acetylcholine and increased aortic calmodulin-1 protein abundance and eNOS activity [69]. The beneficial effects of genistein on blood pressure and endothelial dysfunction may be due to increased eNOS activity associated with increased calmodulin-1 expression and declined O_2_ (^−^) generation. The effects of genistein on hypertension are shown in Table 4. The possible therapeutic effects of genistein on CVDs are shown in Figure 2.

## 4. Conclusion

*In vitro* and *in vivo* studies reported potential therapeutic effects of genistein on cardiovascular disorders including improving cardiac dysfunction, ischemia and reperfusion of heart, decreasing cardiac toxicity, modulating lipid profile, and lowering the blood pressure which are reviewed in the current article. The preventive or prophylactic effects of genistein on cardiovascular disorders may be due to anti-inflammatory, antioxidant, and immunomodulatory effects. Although the effect of genistein on CVD is well shown in the reviewed studies, while further standard clinical trials and meta-analyzing the previous results are needed to be performed.

## Figures and Tables

**Table 1:** The effects of genistein on cardiac dysfunction and ischemia/reperfusion.

Study Design	Doses	Route of administration	Effects	Ref
H9c2 embryonic rat cardiac cells	(0, 50, 100, 150, 200) *μ*mol/L	Expose	↓CoCl2-induced cell death ↓HIF-1*α* expression ↓CoCl2-induced apoptosis ↑Bcl-2/Bax ratio	[18]

Human umbilical cord blood (HUCB)	10^10^–10^−5^ *μ*mol	Expose	↑ECFCs' proliferation and migration ↑Expression of *α*-parvin, F-actin, ILK ↑Phosphorylation of the signaling of ERK 1/2 ↑ECFCs (GS-ECFCs) ↑Proliferation of the Cells ↑Angiogenic cytokines ↑Neovascularization ↓Myocardial fibrosis ↑Cardiac function ↑Regenerative potential in ischemic tissues	[19]

H9c2 cardiomyoblast cells	10^−10^–10^−5^ M	Expose	↓Caspase-3, caspase-8, caspase-9, and Bad ↑Phosphor (p)-Akt, p-Erk1/2, and p-Bad. ↑Erk1/2, Akt, and NF *κ* B proteins	[20]

H9c2 cells	10 *µ*M	Expose	↓Damage of H9c2 cells ↑Cells growth ability ↓Apoptosis ↓Inflammatory response ↓Myc expression ↓P-P38 MAPK and p-JNK expression	[21]

Cardiomyoblasts (H9C2)	10 Pm–100 nM	Expose	↑Endothelial NOS‑dependent NO by the modulation of intracellular signaling related to p38MAPK, ERK1/2, and Akt	[22]

Human Heart cells	5 mg/kg	Expose	↓PICR and P/Ht ↑Dysfunction ↓MNCX ↑MKATP channels	[23]

Cultured cardiac fibroblasts (CFs)	10,50,100 *μ*M	Expose	↓TGF*β*1-induced proliferation ↓production of collagen ↓Myofibroblast transformation ↓TGF*β*-activated kinase 1 (TAK1) ↑Antifibrotic effects	[27]

Culture of ventricular myocytes from neonatal rats	70 *μ*M	Expose	↓ANF ↓Fos ↓MLC-2 ↓(MAP) kinases ↓GTP	[28]

Rats	250 mg/kg	Injected once daily for 2 days	↑Contractility ↑Cardiac output ↑Ischemic tolerance ↑Mean (SEM) recovery of contractility and cardiac output ↓Mean glucose transporter protein 4	[24]

Rats	0.1, 0.2 mg/kg	Subcutaneous injection	↓Heart weight to body weight ratio ↓Left ventricular mass ↓1-OH proline and oxidative stress ↓ Myocardial fibrosis	[25]

Rats	5 mg/kg or 3 mg/kg	Intraperitoneal	↑SR Ca leak ↑Diastolic contracture	[26]

C57/BL6 mice	40 mg/kg/day for 7 weeks	Gavage	↑Cross-sectional area of cardiomyocytes ↓AKT/GSK-3*β* signaling and MAPK (JNK1/2, P38, and ERK1/2) ↑Collagen volume fraction ↑ANP ↓BNP ↑*β*-MHC ↓CTGF ↓Fibronectin, collagen I*α*, collagen III ↓TGF-*β*1 ↓Vimentin mRNA ↓MAPK (JNK1/2, P38, and ERK1/2)	[37]

Rats	1 mg/kg/day	Injection	↓Right ventricular ↓PH-induced pulmonary vascular	[30]

Pigs	0.075 mg/min	Infused	↑Coronary blood flow ↑Phosphorylation of NO synthase ↑NO production through p38 MAPK, ERK 1/2, and Akt pathways ↑Coronary vasodilation ↑Release of NO	[31]

Rats	(0.25, 0.5, 1.0, 1.5, 3, and 5 mg kg)	I.V., 5 min after coronary artery occlusion	↓Necrosis of myocardia ↓MPO activity ↓Serum CPK activity ↑Myocardial contractility ↓Ventricular arrhythmias ↓TNF-alpha and blunted ICAM-1 ↓Inflammatory response	[32]

In 22 postmenopausal patients	54 mg/day	One-year genistein dietary supplementation	↑LA area fractional change ↑LV ejection fraction ↑LA remodeling	[33]

Human	—	Dietary intake	↓NF-kappa B ↓Akt signaling ↓Direct antioxidant action ↓Estrogen and androgen-mediated molecular pathways	[34]

Rabbits	0.2 mg/kg/day	Subcutaneously	A single dose of genistein had no protective effect	[35]

Mice	600 mg/kg	Were fed	↑Echocardiographic changes in function ↑LVID ↑Whole heart surface area ↑Cardiac GLUT4 protein expression ↑GLUT4 protein expression	[36]

**Table 2:** The effects of genistein on cardio toxicity.

Study Design	Doses	Route of administration	Effects	Ref
Adult rat ventricular myocytes	20 microM	Dietary intake	↓Glucose toxicity-induced cardiac mechanical dysfunction	[38]

Guinea pig model	—	Chronic: daily subcutaneous injection for 10 days Acute: intraperitoneal injection 1 hour before endotoxic shock	↑plasma nitrate concentration ↑cGMP ↓APD	[39]

Mice	5 mg/kg	Injection	↓Expressions of IL-6, IL-8, TNF-*α* ↑Protein expressions of HO-1, Nrf-2, NQO1 ↓Apoptosis	[40]

Diabetic rats	300 mg/kg/day	Administered po for 24 weeks	↓Blood glucose ↓HbA1c ↓C-reactive protein ↓Expression of TNF-*α* ↓TGF-*β*1 proteins ↑Total antioxidant	[41]

Mice	(0.25, 0.5 or 1 mg/kg)	intraperitoneal injection	↓Burn-induced myocardial injury ↑LVEF ↓Lactate dehydrogenase ↓level of creatine kinase ↓Apoptosis ↓Inducible NO synthase ↓NO and superoxide anions production ↑Expression of Hes1 and NICD1 ↓Burn-induced myocardial injury ↓Oxidative/nitrative stress	[42]

**Table 3:** Genistein effects on lipids profile.

Study Design	Doses	Route of administration	Effects	Ref
HepG2 cells cultured	0, 0.01, 1.00, 10.00, and 50.00 *µ*M	*Expose*	↑Total-cholesterol levels ↓HDL-C ↓Apo-A1 ↑Sterol regulatory element binding proteins-2)SREBP-2) at protein and mRNA levels ↑Low-density lipoprotein receptor (LDLR) mRNA levels ↑3-hydroxy-3-methyl glutaryl coenzyme A reductase (HMGCR) ↑Intracellular cholesterol synthesis ↓MRNA levels of (PPAR*γ*) ↓*X* receptor of liver (LXR*α*) ↓LXR*α* and ATP-binding cassette transporter A1 (ABCA1) protein levels ↑Intracellular cholesterol levels ↑SREBP-2/LDLR/HMGCR pathway ↓PPAR*γ*/LXR*α*/ABCA1 pathway ↓Cholesterol plasma levels ↑Cholesterol absorption ↓Cholesterol efflux	[43]

Human umbilical vein endothelial cells (HUVECs)	1000 nM	Pretreated with genistein	↓Ox-LDL induced senescence ↓SA-*β*-gal activity and P16 and P21 protein ↑Autophagic flux ↑LC3-II, ↓p-P70S6K, p-mTOR, and P62 level	[44]

Rats	50 mg/kg	Gavaged	↓Triglyceride and HDL level more than LCG or LC ↓TNF-*α*, and IL-6	[45]

Rats	20 mg/kg	Gavage	↓Levels of malondialdehyde (MDA) ↓TNF-*α* ↑Superoxide dismutase activity ↓Luteinization ↓Inflammatory cytokines	[46]

Rats	10 mg/kg/day	Expose	↓LDL cholesterol ↓Total cholesterol levels	[47]

Rats	600 mg/kg diet	In the diet	↑Plasma insulin level ↓HbA(1C) level ↑Glucokinase level ↓Hyperglycemia and Glucose-6-phosphatase	[48]

Rats	200 ppm	A 4-week feeding study	↓Serum cholesterol ↑Total and trabecular bone mineral density	[49]

Rats	10 mg/kg	Subcutaneous administration	↓Total cholesterol levels ↓Lipoprotein cholesterol levels ↑TT levels ↓Serum cholesterol levels ↑Serum triglycerides	[50]

Hamsters	2 g/kg	Diet	↓Hyperlipidemia ↓Triglyceride and LDL cholesterol ↓Malondialdehyde ↓Liver and plasma cholesterol ↑Hepatic LDL receptor ↑ *α* and *β* mRNA receptors of estrogen	[51]

Volunteers human	250 *μ*mol/L	Was dissolved in dimethyl sulfoxide injection	↓Agonist-induced platelet aggregation ↓Collagen- and ADP-dependent platelet activation ↓Coronary atherosclerosis ↓Platelet activity	[52]

Human	0.5–2.5 microM	Subjects consuming	↓Lipid peroxidation of HDL ↑Conjugated dienes ↓Alterations of apoprotein structure	[53]

Women	90 mg/day	Supplement for 12 week	↓ Triglyceride levels ↓Total cholesterol ↓Ldl	[54]

Postmenopausal women	54 mg	PO for 24 month	↓Fasting glucose ↓HOMA-IR and insulin ↓Homocysteine and Fibrinogen	[55]

Postmenopausal women	60 mg/day	Genistein or placebo capsule	↓Total cholesterol, LDL-C, and TG levels ↑HDL-C levels ↑Expression of LDLR, LXR*α*, and ABCG1 ↓Cholesterol	[56]

**Table 4:** The effects of genistein on hypertension.

Study Design	Doses	Route of administration	Effects	Ref
Postmenopausal rat monocytes, aortic vascular cell, and osteoblasts cultures	10 nM or 1 *μ*M	Exposed	↓Cell adhesion molecules ↓Expression of intercellular adhesion molecule 1 ↓Osteoblastic ↑Antiosteogenic action ↑Bone cells ↑Alkaline phosphatase activity	[57]

Rats	10 microM	Exposure	↓Contractions ↓Acetylcholine and A23187-induced relaxation	[58]

Rats	50 micromol/l	Exposed	↓Expressions of the angiotensin II (Ang II) type 1 (AT1) receptor ↓p22phox NADPH oxidase subunit ↓Angiotensin II-induced superoxide ↓Nitroblue tetrazolium ↓Nitrotyrosine formation ↑Angiotensin II ↓P22phox and AT1 receptor expression	[59]

Rats	0.1–30 micromol/L	SMC were cultured in dishes	↓Proliferation of natural and PDGF-BB-induced SMC ↓Atherosclerotic cardiovascular diseases	[60]

Rats	1 mg/kg/day	Daily injection of genistein	↓Right ventricular pressure ↓Pulmonary vascular remodeling ↓50% in human pulmonary artery smooth muscle cell proliferation ↓Hypertrophy of right ventricular ↓Hypertrophy of ventricular myocyte in neonatal rat	[30]

Wistar rats	1 mg/kg/day	Feed a diet	↓Blood pressure Restored PKC-*β*II, eNOS, and ACE expression Preserved ultrastructural integrity of renal	[61]

Rats	20,80,200 *μ*g/kg	Subcutaneous injection for two weeks	↓Speed of tricuspid regurgitation ↓Ventricular hypertrophy index ↓Pulmonary artery stenosis ↓Smooth muscle proliferation ↓Hypertrophy of right ventricular ↓Hypertrophy of myocardia ↓Pulmonary artery pressure	[62]

C57BL/6J mice	600 mg/kg food	Genistein-containing diet 1 month	↓Elevated serum insulin ↓Serum glucose ↑Insulin to glucose ratio ↑Isolated aortic rings in both sex ↑Vascular function	[63]

In vivo rats	1 mg/kg	Injection	↓Contractile response ↓Hyporesponsiveness to NA ↑Nitrite plasma levels ↑INOS expression ↓Long-term hypotension ↓Hypotension and vascular alterations ↓NO overproduction ↑Tyrosine kinase inhibitor effect	[64]

Rats	10 nM	Exposed	↑Antiaggregatory action ↓Platelet aggregation ↑Antiplatelet activity	[65]

Rats	Low-dose (2/5 *µ*g/kg) and high-dose (25 *µ*g/kg)	Subcutaneous treatments	↓Angiotensin II (46%)	[66]

Rats	Low-dose (20 *µ*g/kg) and high-dose (80 *µ*g/kg)	—	↓Phosphorylation of tyrosine ↓Renal arteries contraction ↓Elevated mean pulmonary arterial pressure ↑HO-1 expression ↓Pulmonary arterial hypertension ↑HO-1 in the lung tissues	[67]

Rats	Normal amounts of genistein in diet (0.06% (wt/wt))	Soy-based diet	↓Hypertension sensitive to NaCl in young ↓Stroke-prone SHRs in male ↓Insulin Plasma ↓Insulin resistance in SHR-SP in NaCl rich diet ↓Cholesterol Plasma ↓Triglycerides	[68]

Rats	(Wistar–Kyoto 10 mg/kg/day); SHR-genistein-faslodex (2.5 mg/kg/day)	Vehicle containing	↓Systolic blood pressure ↑Aortic calmodulin-1 protein ↑NOS activity ↓O_2_ (−) production ↓Elevated blood pressure ↑ENOS activity ↓O_2_ (−) generation	[69]

## Data Availability

The data used to support the findings of this study are included within the article.
